# Improvements in Orientation and Balancing Abilities in Response to One Month of Intensive Slackline-Training. A Randomized Controlled Feasibility Study

**DOI:** 10.3389/fnhum.2017.00055

**Published:** 2017-02-10

**Authors:** Milos Dordevic, Anita Hökelmann, Patrick Müller, Kathrin Rehfeld, Notger G. Müller

**Affiliations:** ^1^Department of Neuroprotection, German Center for Neurodegenerative DiseasesMagdeburg, Germany; ^2^Institute of Sports Science, Otto von Guericke UniversityMagdeburg, Germany; ^3^Center for Behavioral Brain SciencesMagdeburg, Germany

**Keywords:** balance, slackline-training, orientation, vestibular system, hippocampus

## Abstract

**Background:** Slackline-training has been shown to improve mainly task-specific balancing skills. Non-task specific effects were assessed for tandem stance and preferred one-leg stance on stable and perturbed force platforms with open eyes. It is unclear whether transfer effects exist for other balancing conditions and which component of the balancing ability is affected. Also, it is not known whether slackline-training can improve non-visual-dependent spatial orientation abilities, a function mainly supported by the hippocampus.

**Objective:** To assess the effect of one-month of slackline-training on different components of balancing ability and its transfer effects on non-visual-dependent spatial orientation abilities.

**Materials and Methods:** Fifty subjects aged 18–30 were randomly assigned to the training group (T) (*n* = 25, 23.2 ± 2.5 years; 12 females) and the control group (C) (*n* = 25, 24.4 ± 2.8 years; 11 females). Professional instructors taught the intervention group to slackline over four consecutive weeks with three 60-min-trainings in each week. Data acquisition was performed (within 2 days) by blinded investigators at the baseline and after the training. Main outcomes Improvement in the score of a 30-item clinical balance test (CBT) developed at our institute (max. score = 90 points) and in the average error distance (in centimeters) in an orientation test (OT), a triangle completion task with walking and wheelchair conditions for 60°, 90°, and 120°.

**Results:** Training group performed significantly better on the closed-eyes conditions of the CBT (1.6 points, 95% CI: 0.6 to 2.6 points vs. 0.1 points, 95% CI: –1 to 1.1 points; *p* = 0.011, $\eta_{p}^{2}$ = 0.128) and in the wheelchair (vestibular) condition of the OT (21 cm, 95% CI: 8–34 cm vs. 1 cm, 95% CI: –14–16 cm; *p* = 0.049, $\eta_{p}^{2}$ = 0.013).

**Conclusion:** Our results indicate that one month of intensive slackline training is a novel approach for enhancing clinically relevant balancing abilities in conditions with closed eyes as well as for improving the vestibular-dependent spatial orientation capability; both of the benefits are likely caused by positive influence of slackline-training on the vestibular system function.

## Introduction

Intact balance control is required not only to maintain postural stability but also to assure safe mobility-related activities during daily life (Mancini and Horak, 2010). Approximately 1.5% of healthcare expenditures in European countries are caused by falls, which mainly occur because of impaired balance, aging and cognitive decline (Ambrose et al., 2013); this large number does not take into account any additional indirect costs. Prevention in the earliest stages, already at young age, is hence justified. Balance and strength training is considered to be by far the most efficient intervention for fall prevention (Karlsson et al., 2013) and it can be effective for postural and neuromuscular control improvements; in addition, balance training is considered to be an effective intervention for improvement in static postural sway and dynamic balance in both athletes and non-athletes (Zech et al., 2010). Moreover, both gray and white matter alterations have been reported in young people in response to only six weeks of balance training (Taubert et al., 2010). The optimal interaction between visual, vestibular and somatosensory systems is the key to stability of the body. While the visual factor can be corrected in many different ways, the other two can be best enhanced through optimal training interventions.

Several recent studies have demonstrated particularly beneficial effect of slacklining on balancing abilities in both younger and older populations (Pfusterschmied et al., 2013; Thomas and Kalicinski, 2016), through enhancement in postural control and functional knee joint stability. Although in other studies mainly task-specific effects were found in response to six weeks of slackline training, larger non-task specific effects on postural control could not be found in these studies for only several relatively simple testing assignments, such as one-leg and tandem stance on stable force platform surface (Donath et al., 2013, 2016b); moreover, the amount of training in these studies was limited to approximately only one hour per week. Slackline length in previous studies was set to between 5 and over 15 m, which proportionally decreases the rate of turns per training, limiting thereby the stimulation of the vestibular system and its output pathways mainly to the otolith organs; in other words, important function of semicircular canals and related brain regions might have been underemployed and an additional potential effect overseen (Highstein, 1991; Cullen and Minor, 2002). Earlier research using several other types of balance-training interventions found transfer effects of training on performance in clinical tests of balance, and these tests are considered very important for both diagnostic and therapeutic purposes (Mancini and Horak, 2010). Some questions remain, however, still unanswered: (1) can slackline-training cause such non-specific transfer effects on performance in a comprehensive clinical balance test clinical balance test (CBT) and (2) what component of balancing ability, as assessed by this test, is mainly affected by slackline-training, with the vestibular component being of particular interest here.

The hippocampus and neighboring cortical regions are the main loci where the onset of Alzheimer’s disease pathology occurs (Raskin et al., 2015), followed by their progressive degeneration, and early prevention treatments (in younger age) concerning this problem are strongly encouraged (Brookmeyer et al., 2007). Several previous animal and human studies have pointed towards a strong link between the vestibular system and orientation centers of the brain, considered to be located in the hippocampus and neighboring regions (Stackman et al., 2002; Russell et al., 2003; Brandt et al., 2005; Jahn et al., 2009). These studies found serious deficits in the orientation function of the temporal lobe as a result of disturbed or lost vestibular input. Many other studies also suggested that the vestibular system provides self-motion information which is important for the hippocampus and related brain regions to develop spatial memories; when this input is lost, spatial memory becomes impaired (Smith et al., 2010). Moreover, professionals who intensively make use of their vestibular system during their daily artistic performances, such as ballet/ice(Pfusterschmied et al., 2011) dancers and slackliners, have differently structured temporal brain regions, including the hippocampus, compared to non-professionals (Hüfner et al., 2011). A study by Allen et al. (2004) clearly demonstrated a reduction in vestibular-kinesthetic dependent orientation abilities with aging, by comparing performance of younger and older adults on the triangle completion task; the older adults performed particularly worse on this task when their input was restricted to the vestibular system only (passively pushed in a wheelchair), implying deterioration of this system with aging. A question that remains unanswered here is if an intensive slackline-training can lead to significant improvement in the vestibular system’s function, which can then be beneficial for spatial orientation abilities in a trained person. Therefore, here we wanted to find out whether an especially challenging balance training program (learning to slackline) can also induce transfer effects on cognitive function, namely spatial orientation. The idea behind this assumption was that a) a strong connection between the vestibular system (which is important for balancing) and the hippocampus has been suggested and b) that spatial orientation is a function that is to a great extent supported by the hippocampus (Hitier et al., 2014). We chose intensive slacklining in young adults as an intervention measure under the assumption that if this training is not capable of inducing transfer effects then other, less demanding regimen (such as those typically used to enhance balancing skills in elderly, sick patients) will surely not be able to do so either. In other words, this was a feasibility pilot study, using a young population.

Thus, having in mind the close connection between the vestibular and orientation systems, we asked whether intensive slackline training can improve not only one’s ability to maintain balance but also has transfer effects on the capability to successfully orientate in space. Up to this point we are not aware of any longitudinal studies that investigated whether the vestibular-dependent temporal lobe orientation function can be enhanced through an intervention aimed towards improvements in balancing skills. The goal of this study was to find out whether learning how to slackline over a period of one month can be of benefit for both stability and orientation skills.

## Materials and Methods

### Ethics Statement

This study was carried out in accordance with the recommendations of and was approved by the Medical Faculty Ethics Committee at the Otto von Guericke University (approval number: 156/14). Each participant signed a document of informed consent before the beginning of the study.

### Subjects

Fifty healthy young (18 to 30 years old) subjects were recruited for this study and randomly assigned (without stratification) into two groups, control (12 females and 13 males; mean age = 23.2 years; *SD* = 2.6 years) and training (11 females and 14 males; mean age = 24.4 years; SD = 2.7 years) (**Table 1**). The two groups did not significantly differ in any of the recorded demographic and other characteristics, including age, height, weight, years of education, handedness etc. Physical activity was assessed by asking subjects how many hours they spend on sports weekly on average; all sports were taken into consideration, including jogging, various team sports, cycling etc., but not walking. Participants of both groups were paid the same amount of money for their participation in the study. Sample size and characteristics, as well as the balance-training duration have been justified by several previous slackline- and other balance-training studies (Zech et al., 2010; Pfusterschmied et al., 2013).

**Table 1:** Characteristics of participants.

Characteristic	Training (*n* = 25)	Control (*n* = 25)
Age (years)	24.5 ± 2.7	23.2 ± 2.6
Sex (females)	11 (44%)	12 (48%)
Weight (kg)	69.1 ± 12.5	65.0 ± 10.0
Height (cm)	173.4 ± 9.2	170.3 ± 8.4
Hours of activity (per week)	3.0 ± 1.8	3.2 ± 2.5
Handedness (right)	24 (96%)	23 (92%)
Profession (student)	22 (88%)	23 (92%)
Suffered a small injury (e.g., ankle sprain)	5 (20%)	5 (20%)
Ethnic origin		
		
• European	20 (80%)	19 (76%)
• Asian (Indian)	5 (20%)	5 (20%)
• Arabic	0 (0%)	1 (4%)

Eligible subjects for this study were all those aged from 18 to 30 years who had no previous experience in slacklining or similar activity (i.e., highly demanding balancing activities, such as ballet dancing, rhythmic gymnastics etc.) and normal or corrected to normal vision. Exclusion criteria were injuries to the musculoskeletal system and systemic diseases (e.g., cardiovascular, metabolic, nervous system diseases etc.). Participants were recruited through advertisement in the buildings of Otto von Guericke University in Magdeburg, both at the main and medical campus.

### Study Design

**FIGURE 1:**
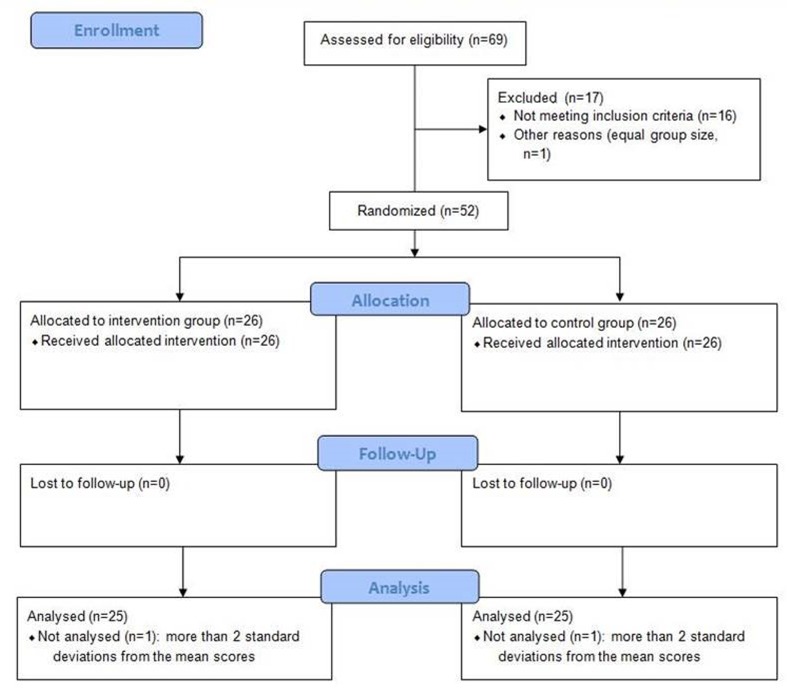
**Study flow diagram**.

Flow diagram of the study is shown in the **Figure 1**. This study was planned and organized as a randomized controlled single-blinded trial with factorial design (factors: time and group). Participants were randomly assigned to the training and control groups using computer-based randomization procedure^1^. The computer-based randomization and assignment of participants to groups were performed by MD (not involved in data collection), with all other investigators blinded to the outcome of the randomization.

The study consisted of measurements at two time points: baseline and one month (±2 days) after baseline. All trainings took place in the movement lab of our institute (German Center for Neurodegenerative Diseases) from February to April 2015.

### Intervention

During this one month period the training group underwent intensive balance training consisting of 12 trainings (three trainings/week with each training lasting 1 h; max. 2 consecutive non-training days) on a 3-m long slackline (“Power-wave 2.0” slackline rack), whilst the control group was instructed to abstain from any type of similar activity; the abstinence from this type of activity was confirmed by control group participants at the post-test.

Trainings were led and supervised by an experienced instructor, whose assignment was to achieve the best possible skill level in the training group participants; content of teaching is shown in the **Table 2**. Minimum requirement to be achieved was set to walking forward two slackline lengths with turn at the end of the first length; each participant must have achieved this minimum requirement to be considered for the analysis, and all participants were successful in achieving this. Each training unit consisted of a 10-min warm up session and 50-min training session. Maximum group size allowed was four participants, so the instructor could dedicate enough time to each trainee. Moreover, the trainings were highly individualized, according to the skill and progression levels of each of the participant. At the end of each training session the instructor collected the information about skill progression, by writing down the achieved skill level of each participant. To do so, the amount of time every participant needed to walk up to four slackline lengths forward, backward, sideways, and turn in between was recorded.

**Table 2:** Contents participants were taught during slackline-trainings; the minimum difficulty level they had to achieve in order to be considered for the final analysis is also presented.

Task	Difficulty levels/Minimum to be achieved
Stable tandem stance	5–10 s/5 s
Stable one-leg stance	5–10 s/5 s
Turn	1–4 times/2 times
Walk forward (with turn for **≥**2)	1–4 lengths/2 lengths
Walk backward (with turn for **≥**2)	1–4 lengths/1 length
Walk sideways (with turn for **≥**2)	1–4 lengths/1 length

The slackline tension was also individualized, so that when standing in the middle and applying a light vertical force (as during walking) the slackline would not get more than several centimeters away from the metal bar located 15 cm underneath. Our goal here was to increase difficulty of training by keeping the slackline slack and thus more unstable, rather than tight and stable, which would otherwise resemble walking on a firm surface. The length of the slackline was also intentionally set to 3 meters; in this way we wanted to achieve a higher rate of turns on the slackline, and thus a higher rate of semicircular canal stimulation. This is in contrast to earlier studies which used moderately to much longer slacklines (5 to over 15 m in length) (Granacher et al., 2010; Pfusterschmied et al., 2013; Donath et al., 2016b), stimulating thereby mainly otolith organs and related central vestibular pathways.

### Tests

All tests were performed by two trained members of our institute and the results of the following sets of measurements were recorded before and after the training:

#### Clinical Balance Test

**FIGURE 2:**
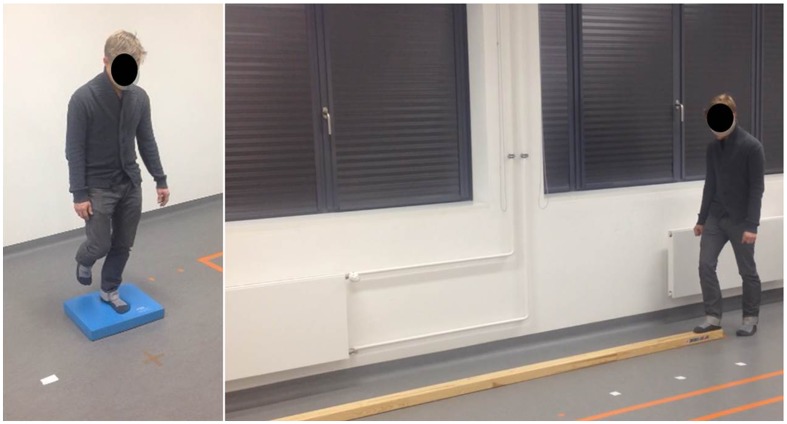
**Examples of clinical balance test (CBT) conditions: unstable surface one-leg stand (left) and balance beam walking (right) conditions**.

Considering that every CBT has its advantages and disadvantages (Mancini and Horak, 2010), this comprehensive test was developed by experts in our institute (DZNE) with the goal to assess different components of patients’ ability to maintain equilibrium, in both standing and gait conditions. Many of the test conditions are consistent with similar comprehensive CBTs (Mancini and Horak, 2010) used in other clinics. The inter-rater reliability of the test (determined with ICC coefficient) is 0.98 ± 0.04 (SEM = 0.003), and its validity is still to be evaluated. The conditions can be briefly divided into standing and walking (**Figure 2**), both of which further contain sub-conditions with open and closed eyes (for detailed list of conditions see the **Table 3**).

**Table 3:** Test conditions of the clinical balance test (CBT).

No.	Condition	Task		Points (min = 0, max = 3)			
				0	1	2	3
1.	Static – stable surface (floor)	Stand with feet together – open eyes					
2.		Stand with feet together – closed eyes					
3.		One leg stance – left – open eyes					
4.		One leg stance – right – open eyes					
5.		One leg stance – left – closed eyes					
6.		One leg stance – right – closed eyes					
7.	Static – unstable surface (pad)	Stand normally (hip width stance) – open eyes					
8.		Stand with feet together – open eyes					
9.		Stand normally (hip width stance) – closed eyes					
10.		Stand with feet together – closed eyes					
11.		One leg stance – left – open eyes					
12.		One leg stance – right – open eyes					
13.		One leg stance – left – closed eyes					
14.		One leg stance – right – closed eyes					
15.	Dynamic	Walk inside the zone (4 m × 30 cm)	Forward				
16.			Turn (90°)				
17.			Backward				
18.		Walk on the line (4 m × 5 cm)	Forward				
19.			Turn (90°)				
20.			Backward				
21.		Walk on the line with feet one after the other (4 m × 5 cm)	Forward				
22.			Turn (90°)				
23.			Backward				
24.		Walk on the beam (4 m × 10 cm)	Forward				
25.			Turn (90°)				
26.			Backward				
27.		Walk on the beam sideways (4 m × 10 cm)	Rightward				
28.			Turn (90°)				
29.			Leftward				
30.		Walk on the line with closed eyes (4 m × 5 cm)	Forward				

Standing conditions include:

• two- and one-leg stance on both stable (floor) and unstable (soft pad) surfaces, with both open and closed eyes

Walking conditions included:

• Walking forwards, backwards, and turning inside a 30 cm wide and 4 m long polygon with open eyes, followed by the same test on a 5 cm wide line as well as on 10 cm wide balance beam.
• Walking forward on 5 cm line with closed eyes.

In total, there are 30 assessment items within this test, 14 of which assess standing and 16 walking; 8 of all measurements are performed with closed eyes. The maximum amount of points that could be collected on the test was 90, with each condition carrying the minimum of 0 and the maximum of 3 points, similar to other comprehensive CBT batteries (Horak et al., 2009). Assessment was based on the subjective opinion of trained assessor who graded postural sway during each of the conditions; to avoid potential differences in subjective opinion between the assessors, each participant was tested by only one assessor at both pre- and post-test. In each of the standing conditions participants were instructed to maintain the required position for 15 s, whereas in walking conditions there was no time requirement and participants were asked to walk at their own pace.

#### Orientation Test (OT)

**FIGURE 3:**
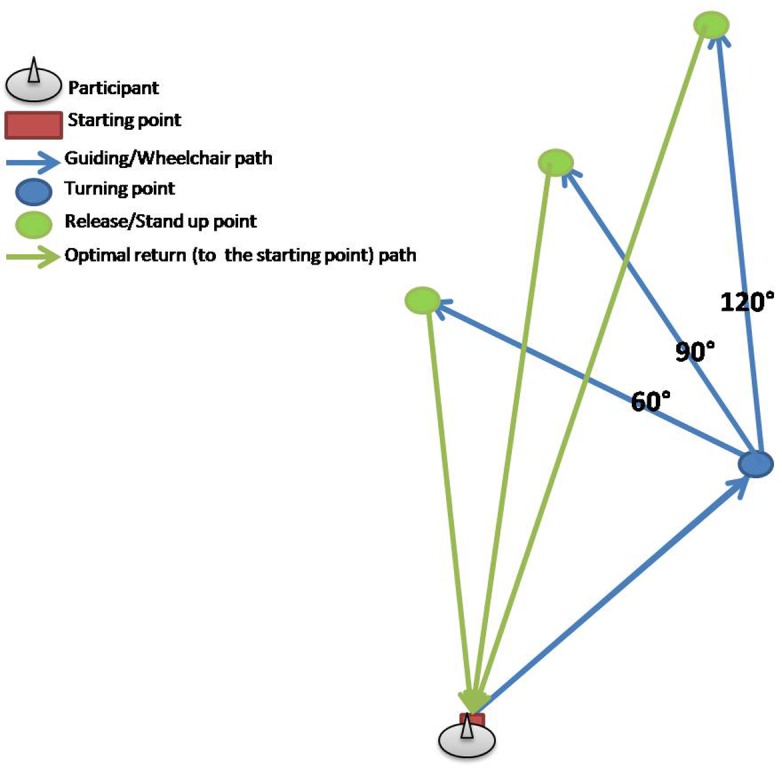
**Example of three triangular paths in right direction used for the orientation test (OT); blue arrows mark two sides of respective triangular paths along which participants where guided or pushed in a wheelchair, whereas green arrows show the optimal route for walking back to the starting point from the respective release/stand up point**.

**FIGURE 4:**
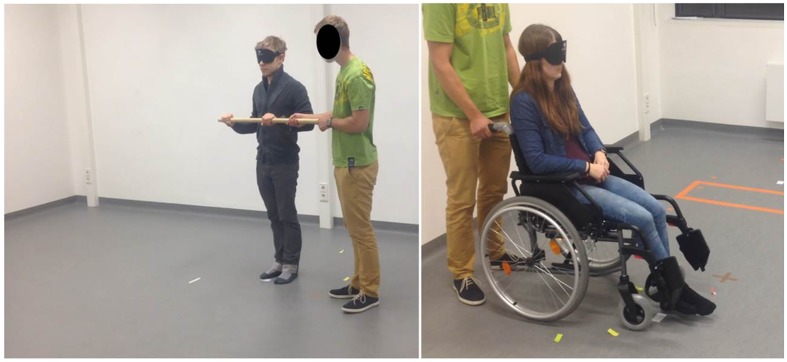
**Examples of OT conditions: guided walking (left) and wheelchair sitting (right) conditions**.

Orientation test was a modified version of the test described by (Allen et al., 2004), whereby the only modification was the inclusion of only three conditions (turning angles) from this study, due to time and space limitations. In brief, six triangular paths were marked on the floor of a room, three in the left and three in the right direction, giving thus three pairs of triangular paths. Lengths of the segments of each triangular path as well as the turn angles between the segments of the triangles are presented in the **Table 4**, with examples of the polygon and test conditions shown in the **Figures 3** and **4**. The test consisted of two conditions: active-walking and passive-wheelchair.

**Table 4:** Length of segments and turning angles of triangular paths in the orientation test (OT).

Direction	Turning angle (°)	Segment 1 (cm)	Segment 2 (cm)	Segment 3 (cm)
Right	60		203	201
	90	203	196	286
	120		250	377
Left	60		203	201
	90	203	196	286
	120		250	377

In the active-walking condition, while being guided on foot, the participant’s movement was controlled by leading him or her along two sides of the triangular path as he or she held onto a wooden bar. The passive-wheelchair condition included transport along the same routes with the use of a standard wheelchair with attached footpads.

Each participant was walked (active) and pushed (passive) only once along each of the paths, giving thus 12 trials per participant in total (3 to the left and 3 to the right, times 2 conditions).

Once the participant was walked/pushed in the wheelchair along two sides of each triangle, his or her task was to walk along the third one, back to the starting point, using thus the shortest possible way back; that is, the participants were instructed not to walk back along the two sides that were used to bring them to the drop-off point, but to use the shortest possible way back to the starting point instead, which is actually always the third side of the respective triangle.

The main outcome variable was the distance error on each trial, which was assessed by marking the participant’s stopping point with adhesive dots on the floor and later measuring the distance from that stopping point to the starting point, from which the respective movement was initiated. The dots were placed on the floor exactly between the feet, aiming thus for the center of pressure, by second assessor, so that the first assessor could focus on giving instructions and guiding participants. After each trial participants were led or pushed back from the stopping point to the starting point, which was for the whole test at the same location, so the next trial could begin.

For the whole duration of the test participants were blindfolded in a quiet room and thereby could not use any visual nor auditory cues that might help them in finding their way back to the starting point. It can thus be assumed that the only cues they could use were somatosensory and vestibular in the active-walking condition and vestibular only in the passive-wheelchair condition.

### Outcome Variables and Data Analysis

Pre-specified primary outcomes were improvement in score (in points) on the CBT and decrement in average error distance (in cm) on the orientation test (OT).

Data were analyzed with MatLab (Mathworks, USA) and SPSS (IBM, USA) software. Statistical analysis included paired *t*-tests for within group analyses and repeated-measures-ANOVAs with time and group as factors for between group and interaction effects analyses. The significance level was set to α = 0.05. The descriptive results are shown as mean ± standard deviation; in addition, effect sizes ($\eta_{p}^{2}$) and 95% confidence intervals of change are reported; the effect size magnitude of ≥0.01 indicated small, ≥0.059 medium and ≥0.138 large effects(Cohen, 1988; Donath et al., 2013). All of the datasets were checked for normal distribution and homogeneity of variance before running parametric tests.

## Results

Final analysis included 25 participants in each group. Two participants (one from each group) were not considered for the analysis because of major outliers, reaching more than 2 standard deviations away from the mean score of all participants. All subjects were recruited from December 2014 until March 2015 and their characteristics are shown in the **Table 1**.

### Clinical Balance Test

**FIGURE 5:**
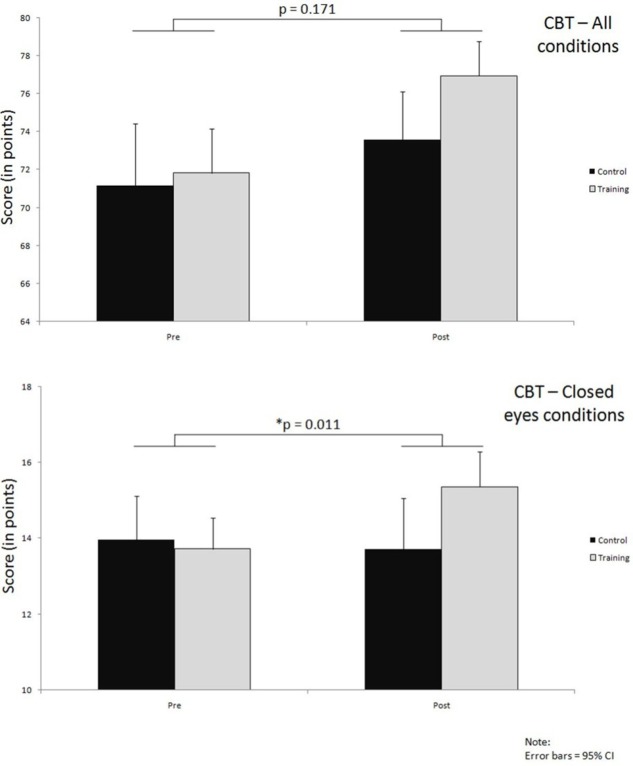
**Improvements over time in both groups on CBT, for all conditions together and closed eyes conditions only; significance levels (*p*) indicate time^∗^group interaction effects**.

**Figure 5** shows both results of the overall test as well as the results for closed eyes condition of the CBT; the respective significance levels are summarized in the **Table 5**.

**Table 5:** Summary of mean improvements over time, interaction effects and effect sizes on CBT and OT; cm – centimeters, pts – points, ^∗^–*p* < α.

Test	Condition	Mean improvements over time (95% CI)		*F*	*p*	$\eta_{p}^{2}$
		Training	Control			
CBT	All conditions	5.1pts (2.5, 7.7pts)	2.4pts (0.4, 5.3pts)	1.93	0.171	0.039
	Closed eyes	1.7pts (0.6, 2.7pts)	0.1pts (–1, 1.1pts)	7.06	0.011^∗^	0.128
	Open eyes	3.5pts (0.9, 6.0pts)	2.5pts (0, 5.1pts)	0.20	0.594	0.004
OT	All conditions	11 cm (2, 19 cm)	–2 cm (–11, 8 cm)	3.46	0.063	0.006
	Wheelchair	21 cm (8, 34 cm)	1 cm (–14, 16 cm)	3.91	0.049^∗^	0.013
	Walking	0 cm (–10, 10 cm)	–4cm (–16, 8 cm)	0.29	0.591	0.001

When overall results are considered, both of the groups demonstrated pre- to post-training improvements. In the training group this improvement was on average 5.1 points (71.8 ± 5.2 to 77.0 ± 4.5) whereas in the control group it amounted to 2.4 points on average (71.1 ± 6.4 to 73.50 ± 4.4). The interaction effect here was not large enough to reach our preset significance level and the effect size was small (*p* = 0.166, $\eta_{p}^{2}$ = 0.039) (**Figure 5**; **Table 5**).

In contrast to the overall test results, when only those conditions were analyzed in which the participants had their eyes closed, a significant interaction effect with medium to large effect size was observed (*p* = 0.011, $\eta_{p}^{2}$ = 0.128), as can be seen from the **Figure 5** and **Table 5**. In these conditions the training group improved (13.7 ± 1.8 to 15.4 ± 2.2) while the control group performed slightly worse on the post-test (13.7 ± 2.6 to 13.6 ± 2.4).

The results from test conditions where participants had their eyes open did not reach significant interaction effect (*p* = 0.594). A learning effect could be observed here, with very similar improvements of about 3 points in both the training and control group (**Table 5**).

### Orientation Test

**FIGURE 6:**
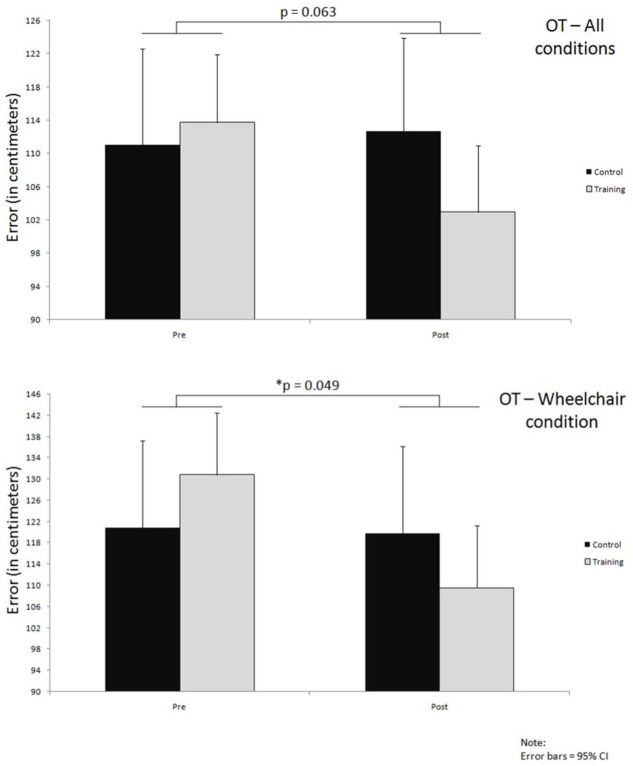
**Improvements over time in both groups on OT, for all conditions together and wheelchair condition only; significance levels (*p*) indicate time^∗^group interaction effects**.

Overall OT results gave a non-significant interaction effect with very small effect size (*p* = 0.063, $\eta_{p}^{2}$ = 0.006) (**Figure 6**; **Table 5**). Errors in the training group decreased by 11 cm (114 ± 68 to 103 ± 62) whereas the error in the control group increased slightly by 2 cm (111 ± 74 to 113 ± 75) (**Figure 6**; **Table 5**).

Further analysis of the wheelchair condition results revealed a much larger improvement in the training group compared to the control group; the training group improved by about 21 cm (131 ± 75 to 110 ± 63) in comparison to a very small 1 cm (121 ± 68 to 120 ± 79) improvement in the control group. This difference in improvements between the two groups that occurred over time led also to a significant interaction effect with small effect size (*p* = 0.049, $\eta_{p}^{2}$ = 0.013) (**Figure 6**; **Table 5**).

Lastly, the condition where participants were walking while actively guided over the polygon did not reveal a significant time x group interaction effect (*p* = 0.591). Within this condition of the OT the training group remained at about the same level of error while the control group performed worse at the post test by about 4 cm (**Table 5**).

## Discussion

The main findings of this study are twofold, both of which are supportive of the a priori hypothesized improvements of vestibular system function in response to intensive balance training.

Firstly, 1 month of intensive balance training during which participants learned how to slackline, led to significantly better performance of our training group participants on the CBT compared to their control counterparts, but only on those measurements where their visual input was blocked, i.e., where they had to balance with eyes closed. The magnitude of the effect of slackline-training here was medium to large. In contrast, on tasks where visual input was not blocked, both groups improved about the same, thus revealing a potential practice effect which might have taken place between pre- and post-test. Considering that the input from three systems involved in balance maintenance is present normally in a moving person (visual, vestibular and somatosensory) (Horak, 2006), it appears from our test results that the vestibular and somatosensory systems were particularly affected by the slackline-training. Secondly and similarly to the previous finding, the training group performed significantly better on the OT compared to the control group, but again only in one condition, namely the passive-wheelchair condition (passively pushed along the designated routes). In this condition the input was intentionally limited to the vestibular system, and the performance thus depended solely on the function of the vestibular system and related brain regions which process this input. Many connections have been proposed to exist between the vestibular system and temporal lobe, in particular the hippocampus, for the purpose of processing these spatial and orientation inputs (Hitier et al., 2014). Once more, the results of the OT used in our study allow us to speculate that vestibulo-hippocampal spatial orientation function has been positively affected by the slackline-training, with a small effect size.

Many earlier studies used numerous diverse approaches to enhance balancing skills in various target groups (Zech et al., 2010; Sherrington et al., 2011). The majority of balance trainings were reported to be successful in improving outcome variables in healthy young (Zech et al., 2010) and elderly (Sherrington et al., 2011; Cadore et al., 2013; El-Khoury et al., 2015) participants, athletes (Hubscher et al., 2010; Boccolini et al., 2013), as well as patients suffering from Alzheimer’s (Ries et al., 2015) and Parkinson’s disease (Sehm et al., 2014), post-stroke patients (Lubetzky-Vilnai and Kartin, 2010) and patients with vestibular disorders (Porciuncula et al., 2012). A literature review pertained to our first finding (stability improvement in closed-eyes conditions of CBT) revealed that similar studies (involving slackline-training) published before suggested large task-specific improvements (standing on slackline) in response to training but only small to moderate non-task specific improvements (for meta-analytical review see (Donath et al., 2016a)). However, these studies used different training and evaluation methodologies; that is, the only non-task specific transfer effects evaluated were postural sway displacement and velocity changes, while participants stood with open eyes on a firm or suddenly perturbed flat surface of a force platform, mostly in one-leg and tandem stance modes. In contrast to these studies, for our analysis outcome from comprehensive clinical balance assessment was used, in which the standing conditions included standing on both and each leg separately (not only one by own choice) in open and closed eyes conditions, on a firm flat but also on a soft, unstable surface. In fact, our main finding here was related to the larger improvement in the closed eyes conditions, which was not even assessed by these studies; for the open-eyes conditions we could also not find any significant effects. Furthermore, our training methodology differed from that applied in previous studies in at least two points: (a) it involved more hours spent on the slackline (around 600 min vs. an average of 380 min in other studies) and was implemented on slacklines of shorter length (3 m vs. 5 to over 15 m in other studies). As we already mentioned earlier, this slackline length was intentionally chosen for the purpose of stimulating semicircular canal function, in addition to that of otolith organs; this important input (Highstein, 1991; Cullen and Minor, 2002) might have been neglected in other training interventions and its effects could hence have been overlooked. Regarding the training intensity, variation in intensity of motor training has already been shown to differentially affect the skill learning and brain structure (Sampaio-Baptista et al., 2014), an effect which could have also contributed to our results. One of previous studies investigated improvements in balancing skills with both open and closed eyes in response to 6 weeks of balance training (Strang et al., 2011). Their results from postural movement measurement were, interestingly, very similar to our results; in the eyes closed condition they noticed a significant improvement while in the eyes open condition no significant change could be observed. The authors argued that this finding was to be expected, because only imposing a constraint during test, such as blockading visual input, would allow the effects of training to emerge. Another study on basketball players also reported improvements in tests with closed eyes in response to a 6-week balance training (Zemkova and Hamar, 2010). Whereas in that study improvements were seen mainly in dynamic balance tests we found them in the static balance tests only, consisting of various conditions on stable and unstable surfaces, which might be due to methodological differences between the studies; that is, the training methods differed and only one dynamic test condition was performed with closed eyes in our CBT, whereas all the other closed eyes conditions of the CBT belonged to the static group. Since the participants improved significantly on the closed eyes conditions, this had to be to the greatest extent within the static conditions. Had, however, our test involved more dynamic conditions with closed eyes, it appears from our results that it would have been reasonable to expect a significant difference in the amount of improvement between groups there as well.

The importance of stimulating both the rotational (semicircular canal function) and the translational (otolith organs) component of the vestibular system becomes obvious and is also crucial for our second finding. Namely, this was to show that the link between the vestibular system and its central vestibular-dependent spatial-orientation brain regions, primarily hippocampal regions (Hitier et al., 2014), can be affected by an adequately designed slackline-training. No previous studies investigated this possibility, making consequently our results novel in that sense. After learning how to slackline, our participants were able to return to the starting position more precisely after being taken away from it in a wheelchair along three different triangular paths. The triangle completion task was already used by many previous studies, mainly to examine the difference between younger and older persons in their ability to navigate in space (Allen et al., 2004; Adamo et al., 2012) or to investigate functions of the medial temporal lobe (Wolbers et al., 2007; Wiener et al., 2011). Consequently, the design of these studies was cross-sectional and no particular treatment was used to improve this ability over time. Our study is the first one to our knowledge to show transfer effects of slackline-training on orientation abilities in young people assessed with this task. Several authors studied rats to demonstrate the importance of the vestibular system for successful orientating in space (Stackman and Herbert, 2002; Russell et al., 2003; Smith et al., 2005). It has been shown that peripheral vestibular deficiency leads to impairments in functioning of the medial temporal lobe in spatial orientation tasks as well as in spatial learning. These impairments are due to alterations in electrophysiological and neurochemical signaling between the two systems. Other previous studies went on further to investigate the importance of the vestibular system for orientation in humans (Brandt et al., 2005; Hüfner et al., 2011; Previc et al., 2014), thereby confirming the findings of animal studies. The structure of the hippocampal formation has been found to be altered in persons who suffer from vestibular deficiency, but also in persons who need to rely heavily on their vestibular system because of their profession, for example ballet dancers. It has even been proposed that vestibular system degeneration might be a significant contributor to development of the Alzheimer’s disease (Previc, 2013). Although our study sample consisted of young and healthy subjects, considering neuroplasticity principles in response to motor task learning over the entire lifespan (Dayan and Cohen, 2011), it is legitimate to hypothesize that similar results could be expected in older populations, particularly as a prevention strategy in those at early stages of dementia. Some studies could not find significant relevant transfer effects of slackline-training in this population (Donath et al., 2016b), but, as discussed earlier, the methodological issues might have contributed to such findings; in our opinion additional research on this topic is required to answer this question.

Therefore, as far as the external validity or generalizability of our findings is concerned, our sample consisted of young and healthy (18–30 years old) subjects, and the results are thus mostly applicable to the same population. Considering, however, that many balance-training interventions can benefit both healthy and diseased populations of various ages in their original form (Taubert et al., 2010; Sehm et al., 2014), it is reasonable to assume that similar interventions to that used in our study could be beneficial for healthy older or even non-healthy older populations, in the direction obtained with our younger sample. It would be of a particular interest for us to see if similar interventions would demonstrate a significant gain in patients suffering from various stages of neurodegeneration, from those with mild cognitive impairment to those with Alzheimer’s disease, in whom the spatial orientation capabilities are considerably reduced (Allen et al., 2004).

There are several limitations of our study which we would like to list here. First, our CBT has not yet been validated; however, many of its items resemble those applied in other validated CBTs and its inter-rater reliability is very high. Another argument against could be that the subjective nature of our postural sway assessment is less accurate than the quantitative assessments at force platforms; most CBTs, however, are subjective opinion-based tests, but are comprehensive and specifically designed to assess various components of balancing abilities, and remain important and valid assessment tools for this purpose (Mancini and Horak, 2010). Our OT comes from the extensively used triangle completion test that assesses orientation abilities; still, only a subset of conditions was applied in our study, in accordance with availability of facilities at our institute; although the error distance measurement has been performed thoroughly, reliability data is still to be provided. Secondly, we did not report any follow up results which would signify a potential of this training to cause eventual retention of the achieved effects over a longer period of subsequent inactivity. Third limitation can be considered the fact that we yet have to show neural correlates of our behavioral improvements, by analyzing pre/post MR data. Finally, our participants performed the training with open eyes; it would be interesting to know whether the same training performed with closed eyes would bring any different results, compared to both the control group and the actual training group, since in this third group visual input would be blocked. We will attempt to successfully deal with these limitations in our future work.

## Conclusion

Our results indicate that 1 month of intensive balance training, through learning how to slackline, is a successful novel approach for enhancing clinically relevant balancing abilities in conditions with closed eyes and simultaneous improvements in vestibular-dependent spatial orientation capability; both of the benefits are possibly caused by positive influence of slackline-training on vestibular system function, and possibly its connectivity with temporal lobe regions responsible for orienting in space, such as the hippocampus. We can highly recommend this method, both its intensity and type, to all young persons who need to improve functioning of their vestibular system, either for the purpose of increasing stability, upgrading spatial orientation abilities or both. Modifying the training protocol could be also potentially of advantage for healthy elderly and those at risk of neurodegeneration of the medial temporal lobe orientation-system, such as in AD, but this is yet to be proven by future studies.

## Author Contributions

MD: Study planning and organization, data collection, data analysis, paper writing, paper revision, paper submission. AH: Study planning and organization, paper revision. PM: Data collection, paper revision. KR: Study planning and organization, paper revision. NM: Study planning and organization, data analysis, paper writing, paper revision.

## Conflict of Interest Statement

The authors declare that the research was conducted in the absence of any commercial or financial relationships that could be construed as a potential conflict of interest.

## Footnotes

1 www.randomizer.org
